# Preface: Science for Regulation and Litigation

**DOI:** 10.1289/ehp.11138

**Published:** 2007-12-13

**Authors:** David Michaels

**Affiliations:** Department of Environmental and Occupational Health, School of Public Health and Health Services, The George Washington University, Washington, DC, E-mail: eohdmm@gwumc.edu

Scientific evidence plays a crucial role in regulation and law, particularly in the area of environmental health. In the courtroom, science helps judges and juries evaluate the disputed facts, whereas in the regulatory arena, science is a vital foundation for effective government decision making. Scientific research is increasingly conducted by parties who desire to influence litigation or regulatory action, and advocates draw readily on litigation or regulation-generated science when it supports their position. Scholars from in the fields of science, law, ethics, and public health met in March 2006 for a conference sponsored by the Project on Scientific Knowledge and Public Policy at The George Washington University School of Public Health to explore the nature of scientific knowledge generated for use in adversarial or advocacy contexts. Participants at the conference “Truth and Advocacy: The Quality and Nature of Litigation and Regulatory Science” presented papers on whether litigation and regulation-generated studies should be judged by the same standards as those conducted outside of the legal and regulatory arenas, how the incentives and intentions that operate in the courtroom and regulatory arenas shape scientific inquiry, and what this implies about the “truth value” of the scientific work.

Funding for the project on Scientific Knowledge and Public Policy (SKAPP) is provided by the Common Benefit Trust, a fund established pursuant to a court order in the Silicone Gel Breast Implant Products Liability litigation. SKAPP staff or planning committee do not provide the funders with advance notice or the opportunity to review or approve any documents or materials produced by the project.

